# Synergy Effect of Plasmonic Field Enhancement and Light Confinement in Mesoporous Titania-Coated Aluminum Nanovoid Photoelectrode

**DOI:** 10.1021/acs.jpclett.3c03150

**Published:** 2023-12-18

**Authors:** Go Kawamura, Daiki Hirai, Shingo Yamauchi, Wai Kian Tan, Hiroyuki Muto, Atsunori Matsuda

**Affiliations:** † Department of Electrical and Electronic Information Engineering, Toyohashi University of Technology, Toyohashi, Aichi 441-8580, Japan; ‡ Institute of Liberal Arts and Science, Toyohashi, Aichi 441-8580, Japan

## Abstract

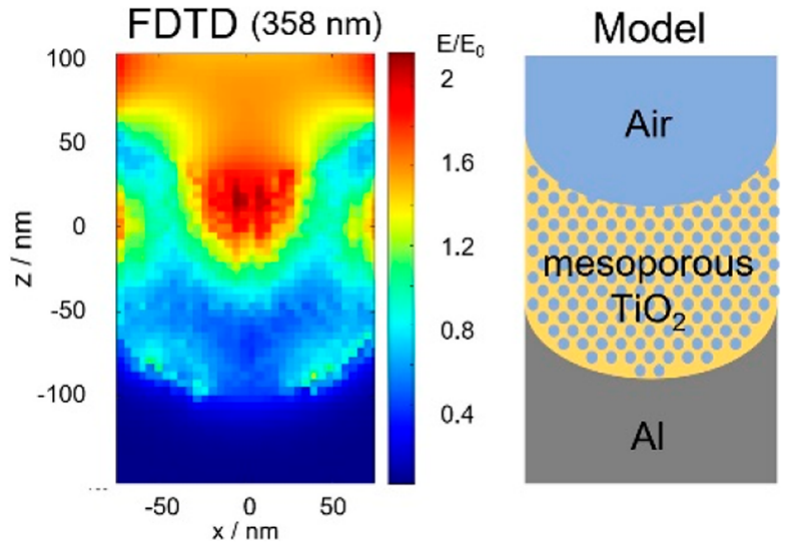

Photoelectrochemical (PEC) water splitting is a highly demanded technology for the realization of sustainable society. Various types of photoanodes have been developed to achieve high efficiency of PEC water splitting. Plasmonic field enhancement and light confinement effects are often adopted to improve PEC performance. However, their synergistic effects have not been studied. In this work, a mesoporous TiO_2_ layer was deposited on an Al plate with a nanovoid array structure, which acts as a photoanode and simultaneously exhibits a light confinement effect and surface plasmon resonance. The solo and synergy effects were investigated through experimental photocurrent measurements and theoretical simulations using the finite-difference time-domain method. The highest improvement in PEC performance was confirmed when the synergy effect occurred.

Solar energy is abundant and sustainable; thus, it is promising to address the current energy and environmental issues.^1^ One strong candidate to overcome the limitations of solar energy, which are fluctuation and intermittence features, is photoelectrochemical (PEC) water splitting. PEC water splitting converts solar energy into storable H_2_ energy, which does not emit greenhouse gases when used. Since the discovery of the Honda–Fujishima effect,^2^ n-type semiconductors, including TiO_2_, have been investigated as efficient photoanodes.^3,4^ Despite the numerous attempts to improve the solar-to-H_2_ conversion efficiency, there is still a large gap between the theoretical maximum and measured values.^5^ Therefore, engineering efficient solar harvesting and utilization remains a great challenge.

PEC performance improvement by localized surface plasmon resonance (LSPR) has attracted considerable attention from researchers. For instance, noble metal nanoparticles showing LSPR are deposited on semiconductor photoanodes to realize performance enhancement. The plasmonic enhancement mechanisms related to light harvesting can be categorized as follows: (1) light scattering effect, (2) light concentration effect, (3) hot electron injection, and (4) plasmon-induced resonance energy transfer.^6−9^ Other effects such as charge separation and local heating also improve PEC performance.^10,11^ The plasmonic enhancement can also be maximized when the wavelengths of incident light and LSPR are matched. As mentioned above, with the enhancement mechanisms being so varied, careful investigation of the phenomena is indispensable for each case. Additionally, developing inexpensive plasmon sources with strong LSPR is required because of the high price of noble metals, such as Au and Ag. The plasmonic materials must also exhibit stability against harsh oxidative situations as water is oxidized with multiple holes on the surface of photoanodes during PEC water splitting.

Adding mesoporosity to the photoanode surface is also often carried out to enlarge its active surface area. For example, TiO_2_^12−14^ and ZnO^15,16^ nanostructured photoanodes with mesoporosity have been widely studied because they are abundant in earth and often show high PEC performance owing to their good light absorption, charge separation, and chemical stability. Besides the large surface area, mesoporous photoanodes possess improved light-harvesting efficiency due to the light scattering and confinement effects.^17−22^ However, the separation of the contributions from large surface area and improved light harvesting, both of which are the results of mesoporosity addition to the PEC performance, is quite challenging. Thus, such a detailed study has rarely been carried out so far.

In this study, an Al plate with nanovoid arrays (Al NVAs) was prepared using the anodization method and employed as an inexpensive plasmonic substrate. A dense and mesoporous TiO_2_ layer was formed on Al NVAs, and the PEC performance was experimentally measured. The PEC performance was improved by adding nanovoid to the Al plate and mesoporosity to the TiO_2_ layer. In the latter part of this article, an electromagnetic field analysis using the finite-difference time-domain (FDTD) method was conducted. With the FDTD simulation, only light harvesting properties can be explored, so that the effect of surface area increase on the PEC performance improvement is excluded; that is, the contributions from large surface area and improved light harvesting to the PEC performance can be separately investigated by the analysis. The nanovoids on the substrate and the mesopores in the TiO_2_ layer offered large field enhancements and were well-coupled to incident light, synergistically contributing to improved PEC performance.

Figure 1A–D shows the scanning electron microscope (SEM) images of Al NVAs prepared with applied voltages of 40 and 120 V for anodization. The void diameters of the samples prepared at 40 and 120 V were 50–100 and 150–350 nm, respectively. Figure 1E summarizes the relationship between the void diameter and applied voltage, including the data of other samples prepared using different applied voltages. It is obvious that the void diameter gradually increased with the applied voltage. Figure 1F shows the diffuse reflectance spectra of the same samples. The samples exhibited extinction peaks in the measurement wavelength region, which could be attributed to LSPR.^23^ The observed peaks were broadened mainly due to the inescapable deviation of the void diameter (seen in panels A–D) because the LSPR wavelength is sensitive to the morphology of plasmonic material.^24,25^ The LSPR peak wavelength had a linear relationship with the applied voltage, at least within the 40 to 120 V range, as shown in Figure 1G. Similar tendency was reported previously,^23^ proving that the observed peaks are due to LSPR. Here, light of wavelength shorter than 300 nm is not contained in the sun light; thus, Al NVAs prepared with applied voltage of 120 V were chosen for the following experiments from a practical application perspective.

**Figure 1:**
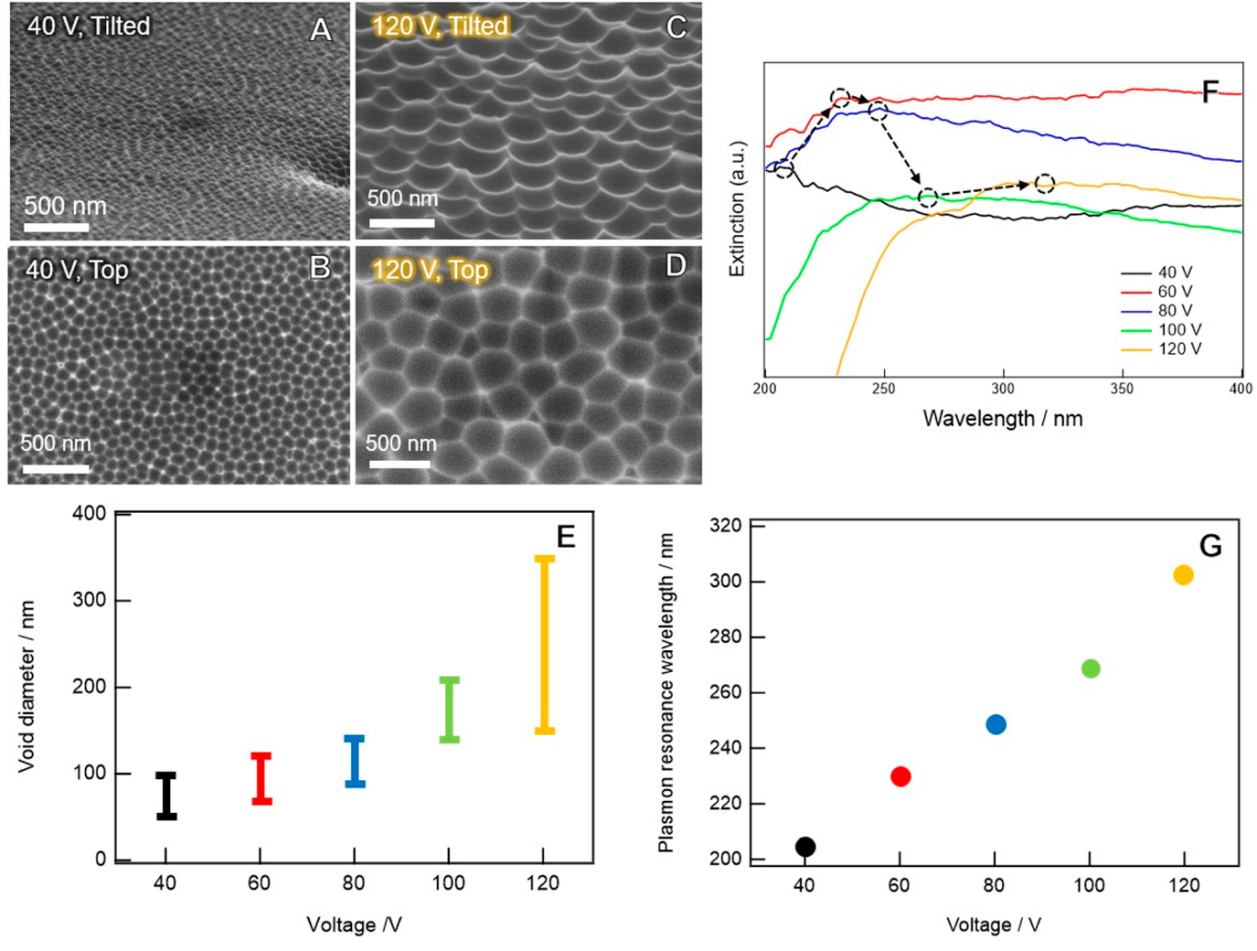
SEM images of Al NVAs prepared with applied voltages of 40 (A and B) and 120 V (C and D). Relationship between the applied voltage and void diameter (E). Diffuse reflectance spectra of Al NVAs prepared under various applied voltages (F). Relationship between the applied voltage and LSPR peak wavelength (indicated by circles in panel F) (G).

Figure 2A,B shows the tilted (45°) SEM images of the dense TiO_2_ layer-coated Al NVAs (Al NVAs/TiO_2_) and the mesoporous TiO_2_ layer-coated Al NVAs/TiO_2_ (Al NVAs/TiO_2_/meso TiO_2_) prepared with an applied voltage of 120 V, respectively. As shown in panel A, the nanovoid structure of the Al substrate is well reflected in the final surface morphology of the dense TiO_2_ layer. The thickness of the dense TiO_2_ was 50–100 nm, as shown in panel A-zoom, where the TiO_2_ layer was intended to be clacked by bending the substrate to measure the thickness by trigonometry. The mesoporous TiO_2_ layer was well formed on Al NVAs/TiO_2_ with a thickness of 130–220 nm. Dense and mesoporous TiO_2_ crystal structures were identified by X-ray diffraction as a single anatase phase (Figure S1).

**Figure 2:**
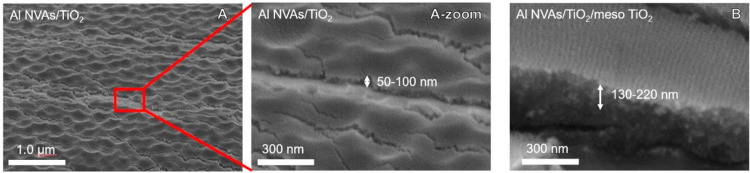
Tilted (45°) SEM images of Al NVAs/TiO_2_ (A) and Al NVAs/TiO_2_/mesoTiO_2_ (B).

The linear sweep voltammogram (LSV) curves obtained using various TiO_2_-coated Al substrates as working electrodes are shown in Figure 3A–C. An Al-flat substrate was used to clarify the merit of the usage of Al NVAs. The dark currents (broken lines) were almost the same and negligible in all samples in Figure 3A,B. Meanwhile, the photocurrent (solid lines) of Al NVAs/TiO_2_ was much larger (3.5 times at 1.23 V) than that of the dense TiO_2_-coated Al-flat substrate (Al/TiO_2_) (see Figure 3A). This is clear evidence that Al NVAs possess higher photoresponsiveness than the Al-flat substrate due to plasmonic field enhancement. The surface area increase due to nanovoid unevenness was only 1.3 times, which also supports the higher photoresponsiveness of Al NVAs. To improve the PEC performance, mesoporous TiO_2_ was coated on the samples to increase the surface area and incorporate a light confinement effect by the mesopores. The results show that the addition of mesoporosity to the TiO_2_ layer leads to a significant enhancement (5.6 times at 1.23 V) in the photocurrent, as shown in Figure 3B. It is worth noting that the dense TiO_2_ layer between the Al substrate and mesoporous TiO_2_ layer played a crucial role in achieving smooth electron transfer at the interface, where no photocurrent response was recorded without the dense TiO_2_ layer. Further performance improvement was observed by depositing a cobalt phosphate (CoPi) cocatalyst to reduce the overpotential for the chemical reaction on the surface and improve the charge separation efficiency, as shown in Figure 3C.^26,27^

**Figure 3:**
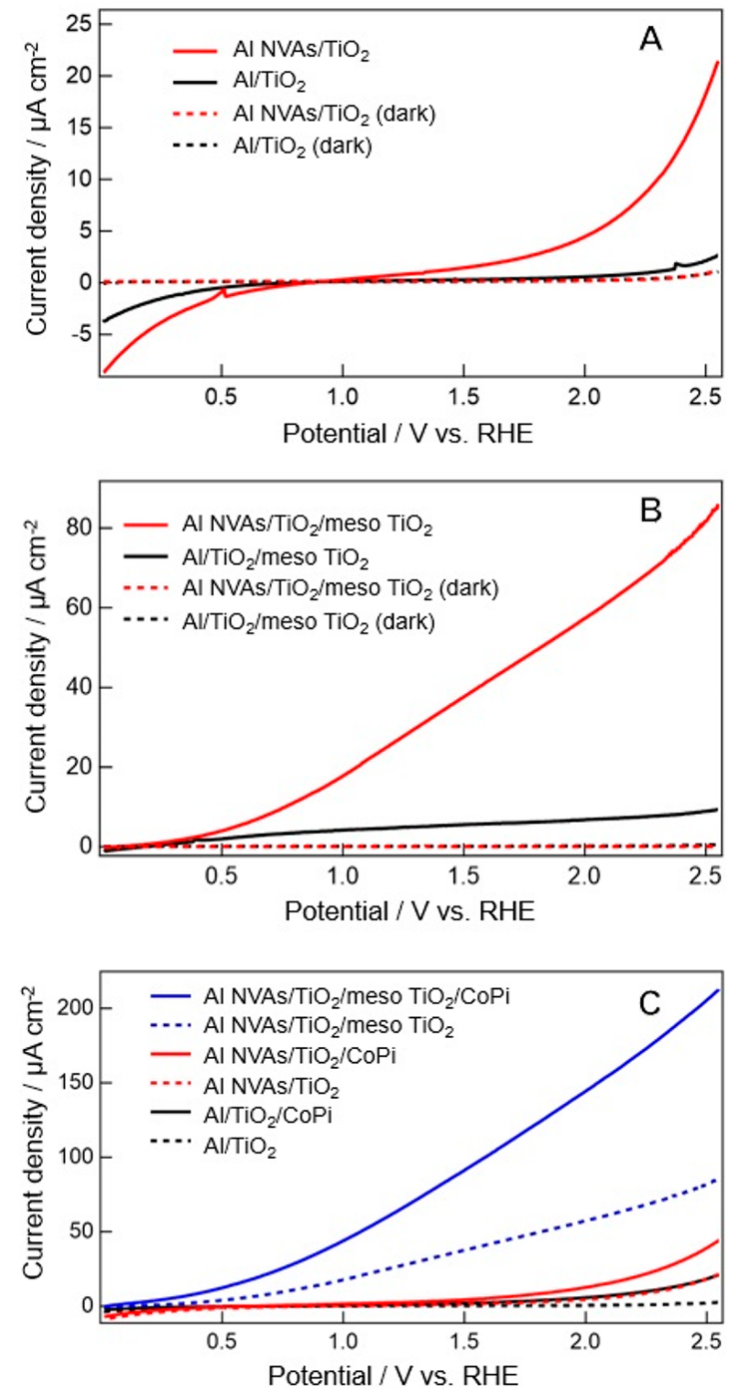
LSV curves of Al/TiO_2_ and Al NVAs/TiO_2_ (A). LSV curves of Al/TiO_2_/meso TiO_2_ and Al NVAs/TiO_2_/meso TiO_2_ (B). LSV curves of Al/TiO_2_, Al/TiO_2_/CoPi, Al NVAs/TiO_2_, Al NVAs/TiO_2_/CoPi, Al NVAs/TiO_2_/meso TiO_2_, and Al NVAs/TiO_2_/meso TiO_2_/CoPi (C).

FDTD simulations were performed to investigate the light-harvesting ability of the prepared photoanodes. Figure 4 shows the simulation results of Al NVAs, Al NVAs/TiO_2_, and Al NVAs/meso TiO_2_. The results of Al NVAs (top line) show that enhanced optical fields are induced near the central *z*-axis of the void without direct contact with the Al surface, which is consistent with the theoretical investigation of plasmonic field enhancement using Mie theory.^28,29^ In our experimental part, the nanovoid arrays were fabricated via anodization. Thus, a plasmonic coupling effect between the voids can also be expected, forming additional plasmonic modes.^23^ In the case of Al NVAs/TiO_2_, clearly enhanced fields are formed in the TiO_2_ layer when the incident light wavelength is between 358 and 400 nm (middle line), which is greater than or almost equal to the anatase band gap energy. Therefore, the TiO_2_ layer is expected to be efficiently excited by the enhanced field, and the generated photocarriers contribute to the photocurrent improvement. Note that the LSPR wavelength is shifted to a longer wavelength region when the dielectric constant of the surrounding medium increases.^30−32^ This explains our case in which a clearly enhanced field was not observed in Al NVAs/TiO_2_ at 325 nm, whereas field enhancement was observed in Al NVAs at the same wavelength. The simulation results of Al NVAs/meso TiO_2_ show a similar tendency to Al NVAs/TiO_2_; i.e., strong optical fields are formed in the TiO_2_ layer in the wavelength region between 358 and 400 nm (bottom line). Here, the simulation model for Al NVAs/meso TiO_2_ was a little bit simplified from the actual structure observed by SEM because too thick and too many mesopores in the TiO_2_ layer made the simulation time too long. It was confirmed that the little difference in the model structures did not affect the final simulation result tendency as shown in Figure S2. On the other hand, the spatial distributions of the strong field in NVAs/meso TiO_2_ vary slightly from them in Al NVAs/TiO_2_. In Al NVAs/meso TiO_2_, the field intensity in the mesopore is higher than that in the other part. This can be explained by the light confinement effect, where the light is repeatedly reflected/scattered at the inner walls of the mesopores, leading to light trapping inside the mesopores.^22^ A large number of photocarriers are generated by the enhanced field in the TiO_2_ layer, whereas the generated carriers (especially holes in this case) must travel to the interface between TiO_2_ and electrolyte. As Al NVAs/meso TiO_2_ has pores inside the TiO_2_ layer, the generated holes are consumed immediately without suffering from charge recombination via traveling in TiO_2_, resulting in a large photocurrent. In other words, the plasmonically enhanced fields are effectively used for photocurrent generation by the light confinement effect of mesoporous TiO_2_. It was further confirmed that the field enhancement was small when mesoporous TiO_2_ was coated on a flat Al substarate (Figure S3). This proved that plasmonic enhancement by nanovoids on Al and light confinement effects by mesopores in TiO_2_ work synergistically. The same FDTD simulations using models with different void diameters of 100 and 300 nm were also carried out (Figure S4). The results showed the same synergy effect as the case using 200 nm void sample, though stronger fields are preferably formed in the longer wavelength region when the sample with larger void diameter was used. This novel synergistic design concept leads to advanced applications of LSPR and light confinement phenomena for future opt-functional devices.

**Figure 4:**
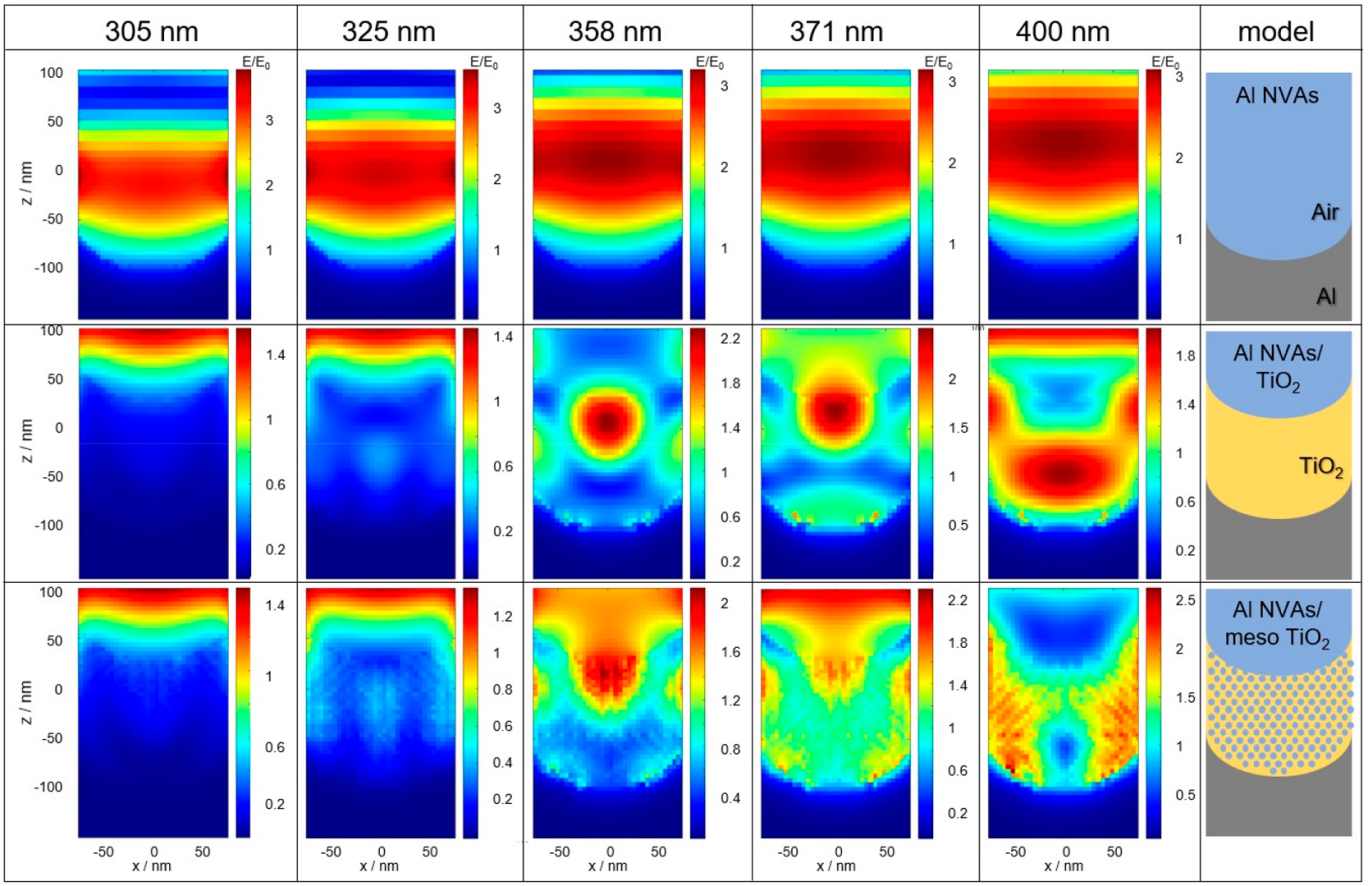
Spatial distributions of field intensity obtained by FDTD simulation at various wavelengths.

We prepared Al NVAs and showed that the nanovoid diameter could be controlled by simply changing the applied voltage during the anodization process. The void diameter had a positive correlation with the LSPR wavelength. A dense and/or mesoporous TiO_2_ layer was then deposited on Al NVAs to fabricate photoanodes. The photocurrent measurement using the photoanodes indicated that the formation of NVAs on an Al plate and the introduction of mesoporosity to the TiO_2_ layer contributed to the PEC performance improvement. A particularly large photocurrent (19.6 times larger than Al/TiO_2_ at 1.23 V) was observed when the working electrode was Al NVAs/TiO_2_/meso TiO_2_, which exhibits LSPR and light confinement effect simultaneously. The FDTD simulation using TiO_2_-coated Al NVAs revealed that enhanced fields were formed by LSPR in the TiO_2_ layer located at the dented area at the wavelength region of 358 to 400 nm, which is well matched with the anatase bandgap energy. When mesoporous TiO_2_ was coated, the enhanced fields were found in the mesopores by the light confinement effect. The photogenerated holes are immediately consumed on the inner walls of the mesopores without traveling because the enhanced fields generate a large number of carriers mainly near the mesopore inner walls, resulting in suppressed charge recombination and large photocurrent. This is the first case to prove that there is a synergy effect between plasmonic field enhancement and light confinement phenomena. The effect plays a crucial role in improving PEC performance.

## Methods

An Al plate (20 mm × 30 mm × 0.3 mm, 99+%, Nilaco, Japan) was washed with acetone and electropolished in an electrolyte composed of HClO_4_ (60–70%, Wako, Japan) and EtOH (1:4 in vol %) at 10 V for 5 min at 10 °C. The backside of the Al plate was masked with a polyimide tape, and the plate was anodized in a 0.5-M H_3_PO_4_ (85+%, Wako, Japan) aqueous electrolyte at 40–120 V for 5–30 min at 3 °C. The porous Al-oxide layer formed on the surface of the Al plate was removed by immersing in an etching solution containing CrO_3_ (98.0+%, Wako, Japan), H_3_PO_4_, and H_2_O (1.8:6.0:92.2 in wt %) at 60 °C for 1 h to obtain Al NVAs.

TiO_2_ precursor sol was prepared by dissolving 5.10 g of Ti(O-n-C_4_H_9_)_4_ (98+%, Wako, Japan) in 13.8 g of ethanol, followed by dropwise addition of a mixture of 6.9 g of ethanol and 0.54 g of 3.6 wt % HCl aqueous solution. After stirring for 1 h, Al NVAs were dip-coated with the TiO_2_ precursor sol at a withdrawal speed of 2 mm/s. The formed dense TiO_2_ layer was finally heated at 450 °C for 15 min. A mesoporous TiO_2_ layer was then coated on Al NVAs/TiO_2_. The precursor sol for the mesoporous TiO_2_ layer was prepared by mixing 12.21 g of Ti(O-i-C_3_H_7_)_4_ (95+%, Wako, Japan), 42.59 g of ethanol, and 3.15 g of Pluronic F-127 (Sigma-Aldrich, Japan) using an ultrasonic bath, followed by dropwise addition of a mixture of 15 g of ethanol and 3.8 g of 36% HCl aqueous solution. After aging for 24 h, the sol was used for spin-coating at 6,000 rpm on Al NVAs. The obtained sample was immediately placed in a freezer at −18 °C for 72 h. It was finally heated at 450 °C for 4 h at a ramp rate of 1 °C/min. A CoPi cocatalyst was deposited using a photodeposition method, where Al NVAs/TiO_2_/meso TiO_2_ was immersed in a mixture of 0.1 M potassium phosphate (pH = 7) and 0.5 mM Co(NO_3_)_2_ and irradiated with ultraviolet (UV) rays (500 mW cm^–2^ at 365 nm, SP-11, Ushio, Japan) for 5 min under a constant 0.1 V.

The surface morphology of samples was observed using an SEM (S-4800, Hitachi, Japan). The diffuse reflectance spectra were recorded using a UV–visible spectrophotometer (V-670, JASCO, Japan). The LSV measurement was carried out by a three-electrode configuration using a Pt coil as a counter electrode and Ag/AgCl as a reference electrode. The electrolyte was 0.1 M potassium phosphate (pH = 7), and an electrochemical analyzer (1280C, Solartron, UK) and a UV-ray light source (500 mW cm^–2^ at 365 nm, SP-11, Ushio, Japan) were used.

Three-dimensional (3D) FDTD simulation was performed using a commercial software package (Lumerical FDTD Solution, Ansys, USA). The 3D models of samples were created based on their SEM observations. The diameter of nanovoids, the thickness of the TiO_2_ layer, and the size of mesopores were 200, 125, and 8 nm, respectively. The boundary conditions were set as periodic for the *x*- and *y*-directions and as perfectly matched layers for the *z*-direction. An unpolarized plane wave source in the range of 350–800 nm was incident to the structures from the top side of the model.

## Supporting Information Available

X-ray diffraction patterns and additional FDTD simulation results (PDF)
Transparent Peer Review report available (PDF)
