# Supplementary material for: Synergy Effect of Plasmonic Field Enhancement and Light Confinement in Mesoporous Titania-Coated Aluminum Nanovoid Photoelectrode
Source: J Phys Chem Lett. 2023 Dec 18;14(51):11691–6. doi: 10.1021/acs.jpclett.3c03150 (PMC10758215; doi:10.1021/acs.jpclett.3c03150)
Supplement: Supplementary file 1 — jz3c03150_si_001.pdf [file jz3c03150_si_001.pdf]

Supporting Information

Synergy Effect of Plasmonic Field Enhancement and Light Confinement in Mesoporous Titania-Coated Aluminum Nanovoid Photoelectrode

Go Kawamura 1,, Daiki Hirai 1, Shingo Yamauchi 1, Wai Kian Tan 2, Hiroyuki Muto 2, Atsunori Matsuda 1*

1 Department of Electrical and Electronic Information Engineering, Toyohashi University of Technology, Toyohashi, 441-8580 Aichi, Japan

2 Institute of Liberal Arts and Science, Toyohashi, 441-8580 Aichi, Japan

AUTHOR INFORMATION

Corresponding Author

* Email: kawamura.go.km@tut.jp

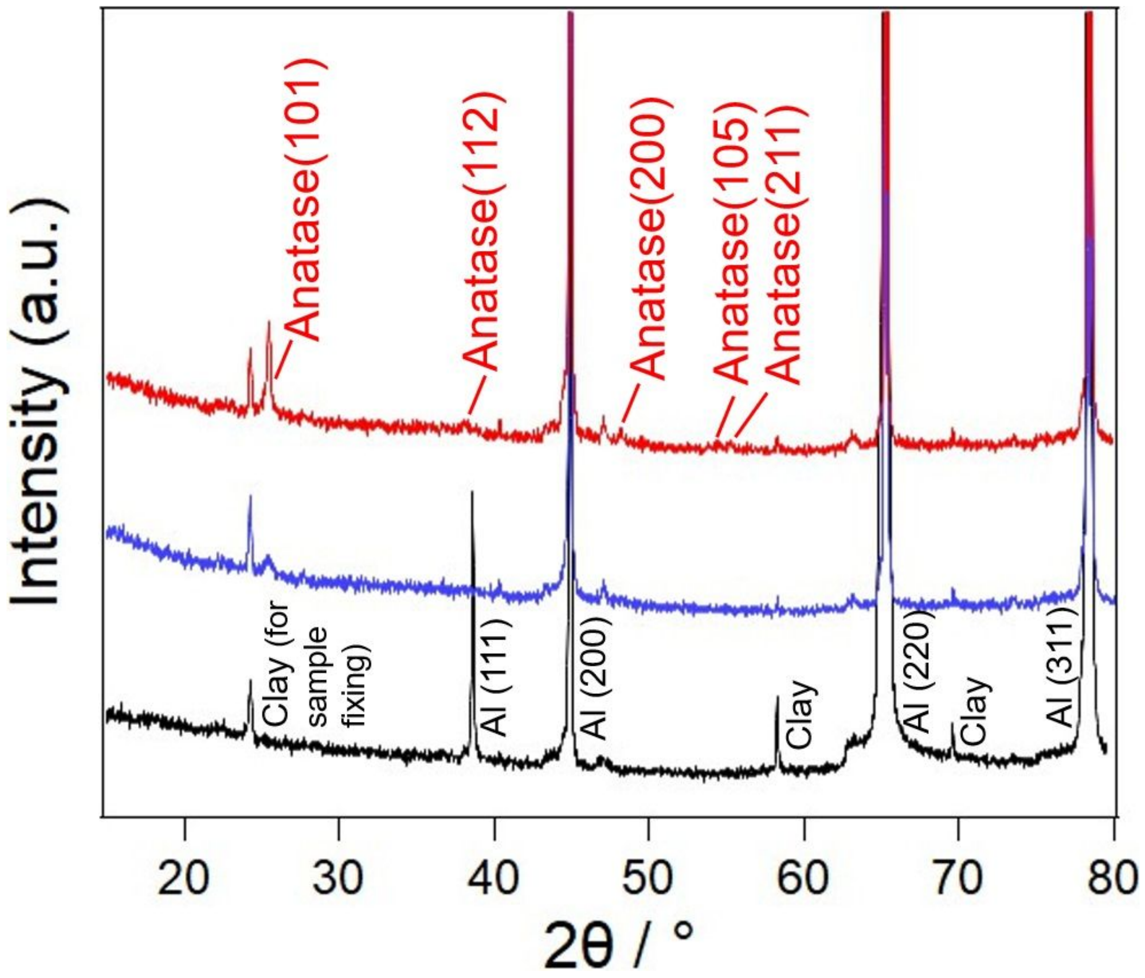

Figure S1. X-ray diffraction pattens of Al substrate (black), dense (blue), and dense+mesoporous TiO_2 (red). An X-ray diffractometer, Ultima IV, Rigaku, Japan (CuKα, 40 kV, 20 mA), was used.

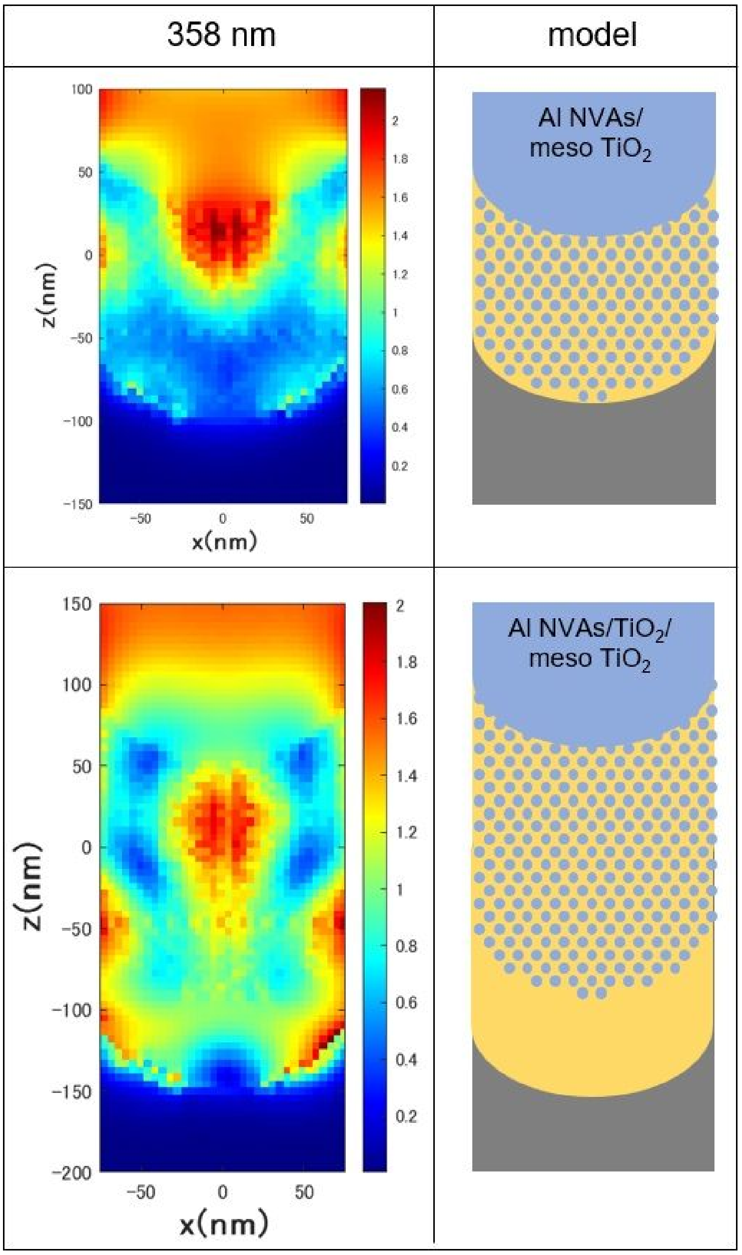

Figure S2. Spatial distributions of field intensity obtained by FDTD simulation with simplified (upper) and actual dimension models (lower).

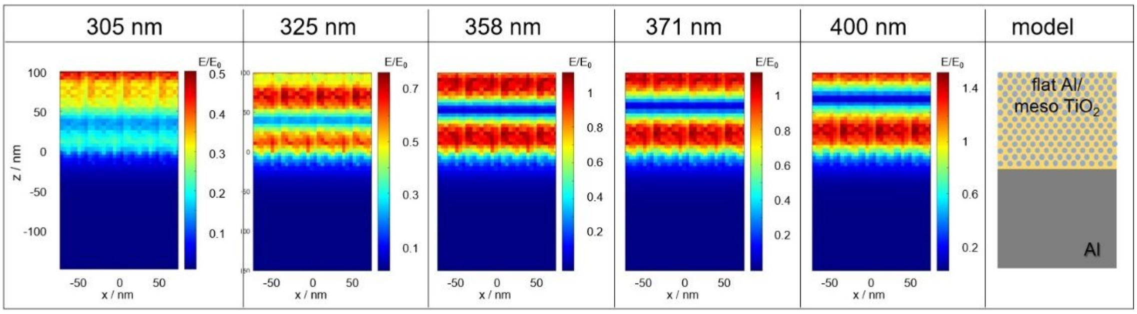

Figure S3. Spatial distributions of field intensity obtained by FDTD simulation at various wavelengths.

A flat Al substrate is employed.

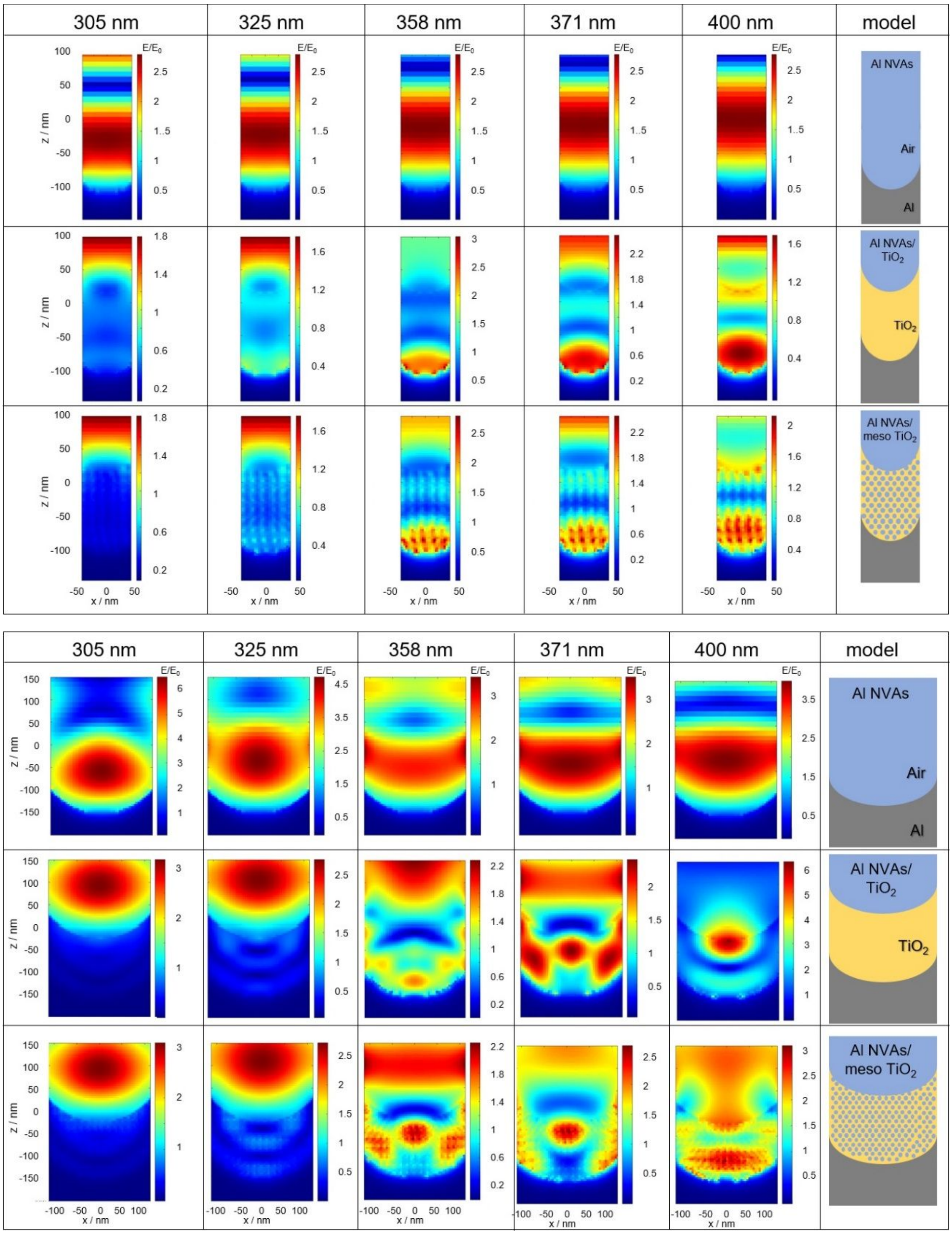

Figure S4. Spatial distributions of field intensity obtained by FDTD simulation at various wavelengths.

The void diameters were 100 (upper) and 300 nm (lower).
