# Supplementary material for: Synergy Effect of Plasmonic Field Enhancement and Light Confinement in Mesoporous Titania-Coated Aluminum Nanovoid Photoelectrode
Source: J Phys Chem Lett. 2023 Dec 18;14(51):11691–6. doi: 10.1021/acs.jpclett.3c03150 (PMC10758215; doi:10.1021/acs.jpclett.3c03150)
Supplement: Supplementary file 2 — jz3c03150_si_002.pdf [file jz3c03150_si_002.pdf]

jz-2023-03150n.R1

Name: Peer Review Information for "Synergy Effect of Plasmonic Field Enhancement and Light Confinement in Mesoporous Titania-Coated Aluminum Nanovoid Photoelectrode"

First Round of Reviewer Comments

Reviewer: 1

Comments to the Author

In this work, a mesoporous TiO_2 layer was deposited on an Al plate with a nanovoid array structure, which acts as a photoanode and simultaneously exhibits a light confinement effect and surface plasmon resonance. The most important highlight of this article is the use of Al as the base of the SPRS effect. The solo and synergy effects were investigated through experimental photocurrent measurements and theoretical simulations using the finite-difference time-domain method. The following comments should be addressed;

1. Provide relevant keywords.

2. “Al plate with nanovoid arrays (Al NVAs) was prepared using anodization method and employed as an inexpensive plasmonic substrate”. It is necessary to provide corresponding experimental data to prove its surface plasmon resonance effect.

3. Can the height of the sample be seen from SEM pictures in Figure 2? We suggest a cross section test.

4. The abstract quality should be improved. In the abstract section, the authors indicated the results of the catalysts. The reason for enhance catalytic performance should be included.

5. Given the topic and scope of the paper, some important references (and their discussion) should be highlighted to broaden the readership such as Materials Today Sustainability 18 (2022) 100118; Adv. Funct. Mater. 2022, 32, 2112738; Chemical Engineering Journal 428 (2022) 131027.

6. It is recommended that the CV of the sample be provided to indicate the difference in the active site of the sample.

7. There are many mistakes in English structure, Scientific language, grammar, and spelling. The whole manuscript should be revised properly.

Reviewer: 2

Comments to the Author

This article presents a theoretical and experimental study of the synergy of plasmonic field enhancement and light confinement for PEC water-splitting. They compared the activity of different photoanodes composed of flat and mesoporous TiO2 films grown on Al nanovoid arrays, showing an improved activity when using NVA/meso TiO2 samples. They explored the light-harvesting properties with FDTD simulations to show that plasmonic enhancement and light confinement effects work synergistically. While the use of plasmonic substrates for PEC is not novel, the results presented here provide further understanding of the source of improvement.

The authors have fully addressed the concerns of the previous review and therefore I suggest that this paper be accepted for publication after tackling the following issues:

1. All figures should have better resolution for publication, especially those containing SEM images (Figure 1A-D, Figure 2)

2. In Figure 1 F, a larger wavelength range should be used to observe the peaks more clearly if possible.

3. Figures 3 and 4 have legends with red underlines (CoPi, meso) that should be removed. The same issue appears in Figures S2 and S4.

Author's Response to Peer Review Comments:

Dear Prof. Editor,

Thank you very much for your effort on our manuscript. We have completed the revision according to the editors and reviewers comments. Hope our manuscript is accepted for publication in JPCL.

Best regards,

Go Kawamura

Reviewer: 1

Recommendation: This paper may be publishable, but major revision is needed; I would like to be invited to review any future revision.

Comments:

In this work, a mesoporous TiO2 layer was deposited on an Al plate with a nanovoid array structure, which acts as a photoanode and simultaneously exhibits a light confinement effect and surface plasmon resonance. The most important highlight of this article is the use of Al as the base of the SPRS effect. The solo and synergy effects were investigated through experimental photocurrent measurements and theoretical simulations using the finite-difference time-domain method. The following comments should be addressed;

1. Provide relevant keywords.

Answer: Thank you for your kind comment. The keywords were added in the abstract page as follows. "KEYWORDS: Photoanode, Localized Surface Plasmon Resonance, Light Harvesting, Finite-Difference Time-Domain Method, Photoelectrochemical Performance"

2. "Al plate with nanovoid arrays (Al NVAs) was prepared using anodization method and employed as an inexpensive plasmonic substrate". It is necessary to provide corresponding experimental data to prove its surface plasmon resonance effect.

Answer: Thank you for your comment. We provide the diffuse reflectance spectra as the experimental data (Fig. 1F) and the FDTD simulation results as theoretical data (Fig. S3 for no LSPR enhancement without nanovoid, and Fig. 4 for LSPR enhancement with nanovoid). We also provide the supportive references (refs. 23, 28, 29) for proving the extinction is due to LSPR. In addition, we added/revised the following sentences to clearly explain that there must be LSPR effect when Al NVAs was employed; "Similar tendency was reported previously,[23] proving that the observed peaks are due to LSPR." at page 5, "This proved that plasmonic enhancement by nanovoids on Al and light confinement effects by mesopores in TiO_2 work synergistically." at page 11.

3. Can the height of the sample be seen from SEM pictures in Figure 2? We suggest a cross section test.

Answer: Thank you for your comment. We calculated the height by trigonometry with 45° tilted SEM images. We have revised the sentences and the caption for Fig. 2 as follows to make it clear; "Figures 2A and B shows the tilted (45°) SEM images of the dense TiO_2 layer-coated Al NVAs (Al NVAs/TiO_2) and the mesoporous TiO_2 layer-coated Al NVAs/TiO_2 (Al NVAs/TiO_2/meso TiO_2) prepared with an applied voltage of 120 V, respectively." at page 6, "The thickness of the dense TiO_2 was 50–100 nm, as shown in panel A-zoom, where the TiO_2 layer was intended to be clacked by bending the substrate to measure the thickness by trigonometry." at page 7, "Figure 2. Tilted (45°) SEM images of Al NVAs/TiO_2 (A) and Al NVAs/TiO_2/mesoTiO_2 (B)."

4. The abstract quality should be improved. In the abstract section, the authors indicated the results of the catalysts. The reason for enhance catalytic performance should be included.

Answer: Thank you for your constructive comment. We have added the following sentence in the abstract section. "The highest improvement in PEC performance was confirmed when the synergy effect occurred.".

5. Given the topic and scope of the paper, some important references (and their discussion) should be highlighted to broaden the readership such as Materials Today Sustainability 18 (2022) 100118; Adv. Funct. Mater. 2022, 32, 2112738; Chemical Engineering Journal 428 (2022) 131027.

Answer: Thank you for your recommendation. The papers are all interesting and "Materials Today Sustainability 18 (2022) 100118" is included in Reference as No. 5. The other papers are a little far from our manuscript topic. Maybe we will cite them in our next work.

6. It is recommended that the CV of the sample be provided to indicate the difference in the active site of the sample.

Answer: Thank you for your constructive comment. We tried to measure the CV of the samples, however the data were not stable and reproducible. The reason of the instability is still not clear, and this would be a challenge for our next work. The current manuscript mainly focuses on the optical property of the samples, thus we decided not to include the CV data.

7. There are many mistakes in English structure, Scientific language, grammar, and spelling. The whole manuscript should be revised properly.

Answer: The manuscript was thoroughly edited by Enago, the editing brand of Crimson Interactive Pvt. Ltd under Normal Editing category. Please check again the English quality of our manuscript.

Reviewer: 2

Recommendation: This paper is publishable subject to minor revisions noted. Further review is not needed.

Comments:
This article presents a theoretical and experimental study of the synergy of plasmonic field enhancement and light confinement for PEC water-splitting. They compared the activity of different photoanodes composed of flat and mesoporous TiO2 films grown on Al nanovoid arrays, showing an improved activity when using NVA/meso TiO2 samples. They explored the light-harvesting properties with FDTD simulations to show that plasmonic enhancement and

light confinement effects work synergistically. While the use of plasmonic substrates for PEC is not novel, the results presented here provide further understanding of the source of improvement.

The authors have fully addressed the concerns of the previous review and therefore I suggest that this paper be accepted for publication after tackling the following issues:
1. All figures should have better resolution for publication, especially those containing SEM images (Figure 1A-D, Figure 2)

Answer: Thank you for your kind advice. The resolution of the figures became low only for review process. We surely provide the figures with higher resolution.

2. In Figure 1 F, a larger wavelength range should be used to observe the peaks more clearly if possible.

Answer: Thank you for your kind suggestion. Since we use TiO_2, which absorbs only UV-rays, we would like to show only the meaningful wavelength region. Therefore, the region in Fig. 1F is set to be from 200 to 400 nm.

3. Figures 3 and 4 have legends with red underlines (CoPi, meso) that should be removed. The same issue appears in Figures S2 and S4.

Answer: Thank you for your kind and detailed check. We revised the figures, and now the red underlines were removed.
